# Impact of Acute Pancreatic Injury on Sphingolipid Metabolism in the Salivary Glands

**DOI:** 10.1155/2020/6403482

**Published:** 2020-08-04

**Authors:** Małgorzata Żendzian-Piotrowska, Dominika M. Ziembicka, Bartłomiej Łukaszuk, Krzysztof Kurek

**Affiliations:** ^1^Department of Hygiene, Epidemiology and Ergonomics, Medical University of Bialystok, Poland; ^2^Department of Public Health, Medical University of Bialystok, Poland; ^3^Department of Physiology, Medical University of Bialystok, Poland; ^4^Department of Gastroenterology and Internal Medicine, Medical University of Bialystok, Poland

## Abstract

Acute pancreatic injury can be related to both parenchymal (responsible for exocrine functions) and islet (mainly *β*-cells, responsible for endocrine functions) damage. During embryonic development, both the salivary glands and the pancreas originate from the foregut, which explains many of the observed histological and functional similarities between these two organs. The relationship between several diseases of the pancreas and salivary glands, resulting from morphological and functional similarities, is well established. Sphingolipids constitute a class of biologically active molecules involved in numerous physiological and pathological processes, including acute pancreatitis (AP) and diabetes mellitus. However, the effect of AP on sphingolipid metabolism in the salivary glands remains uncertain. In the presented study, we examined the effect of AP and type 1 diabetes mellitus on sphingolipid metabolism in the salivary glands of rats. We demonstrated that acute pancreatic injury, related to both exocrine and endocrine functions, affects the metabolism of sphingolipids in the parotid, but not submandibular, salivary glands.

## 1. Introduction

The salivary glands of mammals are exocrine glands, the main function of which is saliva production. Both in humans and rodents, three pairs of macroscopic glands—parotid, submandibular, and sublingual—have been described [1]. Interestingly, during embryonic development, both the salivary glands and the pancreas originate from the foregut, which explains many of the observed histological and functional similarities between these two organs [2, 3]. The secretory end pieces of the salivary glands as well as the pancreas are the acini which produce and secrete saliva or pancreatic juice and bicarbonates into the ductal system of each gland [4]. The second resemblance between the described organs is the secretion of the digestive enzyme *α*-amylase in both the pancreas and the salivary glands [5]. In addition to exocrine function, the pancreas also serves as an endocrine gland, stabilizing the blood glucose level by the release of insulin, a hormone produced by beta-cells (*β*-cells) in the islets of Langerhans.

Acute pancreatic injury can be related to both parenchymal (responsible for exocrine functions) and islet (mainly *β*-cells, responsible for endocrine functions) damage. In the presence of the former, the disease called acute pancreatitis (AP) is one of the most common gastrointestinal disorders. The reported annual incidence of AP ranges from 4.9 to 35 cases per 100,000 people [6]. Pancreatic inflammation activates a cytokine cascade, which often leads to the development of systemic inflammatory response syndrome (SIRS) [7]. A prolonged duration of SIRS increases the risk of multiorgan dysfunction syndrome (MODS), a condition associated with a high mortality rate of up to 39% [8].

It has been observed that AP tends to coincide with type 1 diabetes mellitus (T1DM). It was estimated that up to 40–60% of patients with severe AP develop T1DM [9]. On the other hand, the risk of AP increases in only 1% of T1DM patients [9]. The presented data indicate a strong relationship between AP and T1DM considering the common pathogenesis of these diseases. However, the pathogenesis of AP and T1DM has not been thoroughly explained. It is believed that one of its possible factors may be alterations in sphingolipid metabolism, which has been recently proven in both human and animal models [10, 11].

**Figure 1:**
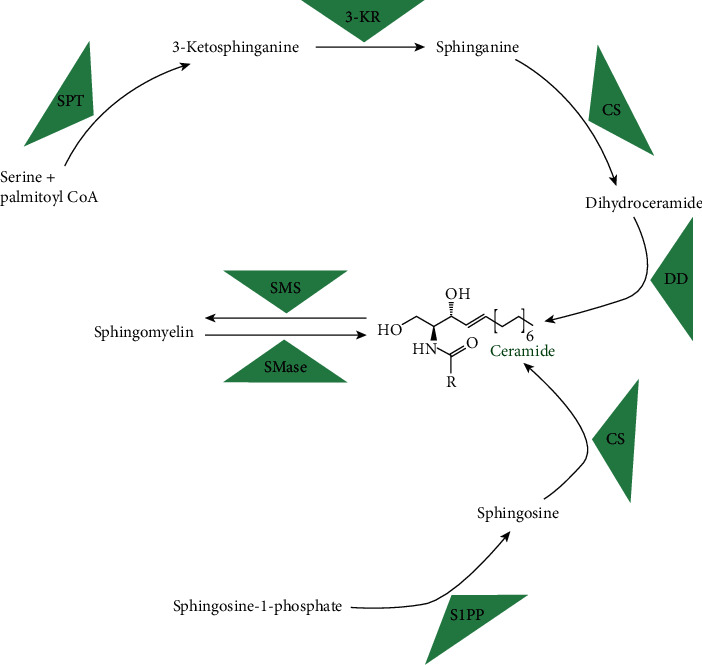
Schematic representation of the sphingomyelin signaling pathway. SPT: serine palmitoyl transferase; 3-KR: 3-ketosphinganine reductase; CS: ceramide synthase; DD: dihydroceramide desaturase; S1PP: sphingosine-1-phosphae phosphatase; SMS: sphingomyelin synthase; SMase: sphingomyelinase.

Sphingolipids (Figure 1) constitute a class of biologically active molecules with a well-established involvement in numerous physiological and pathological processes [12, 13]. Ceramide, which is a central molecule and the main messenger of the sphingomyelin signaling pathway, is a lipid particle able to stimulate and regulate the activities of numerous enzymes [13–15]. To name just a few, (1) an overaccretion of ceramide has been shown to activate PP2A (protein phosphatase 2A), which impairs the insulin signaling pathway by dephosphorylation of threonine and serine molecules in PKB (protein kinase B) [16, 17], and (2) it is postulated that ceramide activates JNKs (c-Jun N-terminal kinases) and IKK (inducer of *κ* kinase), i.e., the enzymes playing a pivotal role in response to stress stimuli and development of inflammation [18].

Intracellular ceramide primarily originates from the de novo synthesis pathway or follows hydrolysis of sphingomyelin [19]. The activation of the sphingomyelin signaling pathway was proven to be involved in the regulation of cell differentiation, proliferation, or even necrosis as well as programmed cell death (apoptosis) [17, 20]. On the other hand, ceramide downstream metabolite sphingosine-1-phosphate (S1P) presents the antagonizing effect on ceramide, forcing it to inhibit cell proliferation, apoptosis, and angiogenesis [14].

Recently, several studies concerning salivary gland lipid metabolism in the course of metabolic diseases have been performed. First of all, Matczuk et al. [21] observed that insulin resistance resulting from a chronic high-fat diet evokes the accumulation of triacylglycerides accompanied by a reduced phospholipid level in the salivary glands of rats [21]. Similar disturbances in lipid metabolism in the salivary glands of rats were observed in the course of streptozotocin-induced diabetes [22]. It was evidenced that the aforementioned changes led directly to salivary gland atrophy and progressive malfunction of the organ. Finally, in the study published by Garbowska et al. [23], activation of the sphingomyelin signaling pathway was observed in the salivary glands of rats in the course of type 1 diabetes induced by streptozotocin. The authors cited above also demonstrated inhibited sphingomyelin signal transduction in the salivary glands of rats subjected to chronic high-fat diet feeding [23]. However, the effect of AP on sphingolipid metabolism in the salivary glands remains unclear. To fulfill this gap of knowledge, we conducted an animal study on cerulein-induced AP and streptozotocin-induced type 1 diabetes to assess the sphingolipid metabolism in the parotid and submandibular salivary glands.

## 2. Materials and Methods

### 2.1. Protocol of the Experiment

The implemented experimental procedures were approved by the Local Ethical Committee for Animal Experiments of the Medical University of Bialystok. The studies were performed on the male Wistar rats obtained from a licensed breeder (age = 6 weeks, weight = 190–240 g). The rats were obtained from the MUB's Center for Experimental Medicine—certified by the Polish official agencies (https://www.umb.edu.pl/o_uczelni/centrum_medycyny_doswiadczalnej). The rodents were provided with the following conditions: stable temperature (21–22°C) and humidity and a 12/12 h light-dark cycle. All rats had unrestricted access to food and water. At the beginning of the experiment, the animals were randomly divided into 3 study groups:

1. Control (C) (*n* = 10)
2. Acute pancreatitis (AP) (*n* = 10)
3. Streptozotocin-induced diabetes (T1DM) (*n* = 10)

The rats assigned to the AP (acute pancreatic) group received cerulein (Sigma-Aldrich) at a dose of 50 *μ*g/kg of body weight, diluted in 1 mL of saline solution and administrated in two intraperitoneal injections with 1-hour interval in accordance with the previously described method [11]. The occurrence of AP was then confirmed by assessing serum amylase and lipase activities 24 hours after the cerulein injections.

The animals from the T1DM (type 1 diabetes mellitus) group received streptozotocin (Sigma-Aldrich) dissolved in citrate buffer, pH 5.4, at a dose of 60 mg/kg of body weight, administrated in an intraperitoneal injection according to the previously described method [15]. The injection was performed upon an overnight fasting 2 days prior to the planned final day of the experiment. The development of type 1 diabetes was then confirmed by measuring fasting glucose level 48 hours after streptozotocin administration.

The animals assigned to group C received intraperitoneally 1 mL of saline solution serving as a placebo.

At the end of the study (48 hours after the cerulein or streptozotocin injection), all rats were anesthetized after overnight fasting by an intraperitoneal pentobarbital injection at a dose of 80 mg/kg body weight and then sacrificed. The samples of both the parotid (PSG) and submandibular (SMSG) salivary glands were excised, immediately frozen between aluminum clamps, precooled in liquid nitrogen, and stored at -80°C until further assays. Blood samples taken from the abdominal aorta were stored at -80°C until further analyses.

### 2.2. Serum Analyses

The activities of serum amylase and lipase as well as the concentration of serum C-reactive protein (CRP) were measured using commercially available laboratory kits (Abbott, USA). The serum glucose level was evaluated with Accu-Chek blood glucose meter (Bayer, Germany). Serum insulin concentration was measured using the chemiluminescence method with a commercial kit (Abbott, USA).

### 2.3. Concentration of Sphingomyelin (SM) in the Salivary Glands

At the beginning of the procedure, tissue samples were pulverized in a precooled aluminum mortar and transferred to tubes containing a solution of methanol and antioxidant (0.01% butylated hydroxytoluene). Then, lipid fractions were isolated in accordance with the method described in the work by Łukaszuk et al. [24]. Sphingomyelin was extracted using thin-layer chromatography (TLC). The gel bands were scraped off the plates upon examining their reference to the standards and transferred into tubes containing pentadecanoic acid used as an internal standard. After transmethylation, fatty acid components of sphingomyelin were analyzed using gas-liquid chromatography (GLC). The Hewlett-Packard 5890 Series II system equipped with a double flame-ionization detector and Agilent CP-Sil 88 capillary column was implemented. Total sphingomyelin concentration was expressed as the sum of individual fatty acid species (in nM/g of the tissue).

### 2.4. Concentration of Ceramide (Cer) in the Salivary Glands

Ceramide content in the salivary glands was assessed using the method described by Garbowska et al. [23]. Briefly, a small portion of the chloroform phase was transferred to a fresh tube containing C17 sphingosine (Avanti Polar Lipids, UK) used as an internal standard. The organic phase containing ceramide was then hydrolyzed at 90°C for 60 minutes in the solution of 1 M KOH in 90% methanol. The obtained sphingosine was subsequently analyzed using the high-performance liquid chromatography (HPLC) technique. N-Palmitoylsphingosine (Avanti Polar Lipids, UK) was applied as a standard for the preparation of the calibration curve. The assessed amount of ceramide was adjusted with respect to the level of free sphingosine in the sample.

### 2.5. Concentrations of Sphinganine (SFA), Sphinganine-1-Phosphate (SFA1P), Sphingosine (SFO), and Sphingosine-1-Phosphate (S1P) in the Salivary Glands

The concentrations of the abovementioned sphingolipids were measured in accordance with the method described by Min et al. [25]. At the beginning of the procedure, prior to the homogenization and ultrasonication of the samples, internal standards (C17 sphingosine and C17 sphingosine-1-phosphate) (Avanti Polar Lipids, UK) were added. Sphingoid bases were converted to their o-phthalaldehyde derivatives and then examined with a HPLC system (ProStar, Varian, Inc., USA) equipped with a fluorescence detector and reversed-phase C18 column (Varian, Inc., OmniSpher 5, 4.6 × 150 mm).

### 2.6. Statistical Analysis

The results were presented as mean ± standard deviation (SD). Statistical differences between the groups (*n* = 10) were assessed using ANOVA with a subsequent post hoc test (Tukey HSD). Statistical significance was assumed at the level *p* < 0.05.

## 3. Results

### 3.1. Activities of Serum Amylase and Lipase: Concentrations of Serum C-Reactive Protein, Glucose, and Insulin (Table 1)

Rats from group AP were characterized by significantly intensified activities of amylase and lipase compared to both groups C (+1.47 fold and +5.67 fold, respectively, *p* < 0.05) and T1DM (+1.64 fold and ~5 fold, respectively, *p* < 0.05). In group AP, serum CRP content was elevated in comparison with groups C (+10 fold, *p* < 0.05) and T1DM (+7.26 fold, *p* < 0.05). Furthermore, we noticed a significant increase in serum glucose in the T1DM group compared to both groups C (+4.1 fold, *p* < 0.05) and AP (+3.55 fold, *p* < 0.05). On the other hand, markedly decreased serum insulin concentration (below the detection level) was observed in T1DM rats compared to both groups C (*p* < 0.05) and AP (*p* < 0.05).

### 3.2. Concentration of Sphingomyelin in the Salivary Glands (Tables 2 and 3)

In both the submandibular and parotid salivary glands, there were no differences in SM content between groups C and AP. However, a significant decrease in SM was noticed in group T1DM compared to C (-28% and -26% for submandibular and parotid salivary glands, respectively, *p* < 0.05) as well as in T1DM compared to AP (-29% and -24% for the submandibular and parotid salivary glands, respectively, *p* < 0.05).

### 3.3. Concentration of Ceramide in the Salivary Glands (Tables 2 and 3)

In the SMSG, no significant differences in ceramide concentrations between any of the examined groups were observed. However, T1DM rats were characterized by a dramatic reduction in ceramide content in PSG compared to both groups C (-84%, *p* < 0.05) and AP (-89%, *p* < 0.05).

### 3.4. Concentration of Sphingosine in the Salivary Glands (Tables 2 and 3)

There were no significant differences in SFO contents in SMSG of groups C and AP. However, in T1DM rats, we noted an increased SFO level in comparison with group C (+33%, *p* < 0.05). In PSG, the concentrations of SFO were considerably elevated in both groups AP (+26%, *p* < 0.05) and T1DM (+23%, *p* < 0.05) compared to group C.

### 3.5. Concentration of Sphinganine in the Salivary Glands (Tables 2 and 3)

In the SMSG, there were no differences in sphinganine concentration between groups C and AP. However, we noticed a significant reduction in SFA content in SMSG of T1DM rats compared to group C (-48%, *p* < 0.05). In PSG, we observed a considerable increase of SFA content in both groups AP (+62%, *p* < 0.05) and T1DM (+138%, *p* < 0.05) compared to group C.

### 3.6. Concentration of Sphingosine-1-Phosphate in the Salivary Glands (Tables 2 and 3)

No differences in S1P concentration in SMSG between groups C and AP were observed. However, rats from the T1DM group were characterized by a significant reduction in the S1P level compared to both groups C (-43%, *p* < 0.05) and AP (-30%, *p* < 0.05). In PSG of T1DM rats, the concentration of S1P was considerably elevated compared to both groups C (+53%, *p* < 0.05) and AP (+24%, *p* < 0.05). In PSG, S1P content was markedly decreased in AP rats (-47%, *p* < 0.05) compared to group C.

### 3.7. Concentration of Sphinganine-1-Phosphate in the Salivary Glands (Tables 2 and 3)

There were no differences in SFA1P content in SMSG between groups C and AP. However, in T1DM rats, we observed a significantly reduced level of SFA1P compared to both groups C (-38%, *p* < 0.05) and AP (-50%, *p* < 0.05). On the other hand, SFA1P concentration in PSG was markedly elevated in both AP (+70%, *p* < 0.05) and T1DM (+30%, *p* < 0.05) groups compared to group C.

## 4. Discussion

Our study investigates the relationship between the salivary gland sphingolipid signaling pathway in acute pancreatitis and streptozotocin-induced experimental diabetes. The pancreas and salivary glands have a similar anatomical structure as a consequence of their analogous embryonic development. It has been demonstrated that both the pancreas and the salivary glands are formed as a result of a controlled sequence of epithelial-mesenchymal mutual interactions. Moreover, both these types of glands serve a comparable function: they produce fluid rich in bicarbonates containing digestive enzymes and other ingredients to be delivered into the gut [26].

The relationship between several pancreatic and salivary gland diseases, resulting from morphological and functional similarities between these organs, is well established. First, it was demonstrated by Kamisawa et al. [26] that chronic pancreatitis of various etiologies frequently impairs the secretory function of the salivary glands, resulting in hyposalivation [26]. What is more, autoimmune diseases, such as autoimmune pancreatitis or IgG4-related disease, lead to progressive fibrosis and functional disturbances in both the pancreas and the salivary glands [27]. In addition, Gokel et al. [28] found that toxic organophosphates induce not only acute pancreatitis (AP) but also parotitis—a state of inflammation affecting the parotid salivary glands [28]. It is worth mentioning at this point that viral infections, especially measles, which typically induce an inflammatory state in the salivary glands, can also affect the pancreas, leading to severe AP with a potentially fatal course [29]. On the other hand, in a study by Benini et al. [30] on lactoferrin concentration in saliva, no difference was observed in the saliva lactoferrin level between healthy subjects and patients with pancreatic cancer or chronic pancreatitis [30].

The relationship between acute pancreatitis and alterations in sphingolipid metabolism has attracted much attention in the recent decade. In the first study concerning the subject, Li et al. [31] observed the activation of sphingosine-1-kinase (SPHK1) enzymatic activity with concomitant increased S1P concentration in blood cells (neutrocytes, monocytes, and lymphocytes) obtained from the peripheral blood of patients with a severe form of AP. Furthermore, the authors demonstrated a positive correlation between S1P level and AP severity. Finally, they revealed reduced expression of SPHK1 to its baseline level during AP restoration, which suggests that S1P concentration could act as a marker of the disease activity [31]. These observations were recently confirmed in a study published by Konończuk et al. Briefly, in the rodent model of AP, it was noted that S1P concentration in pancreatic tissue samples was considerably elevated compared to the control group [11]. A similar increment was observed in the centrifuged plasma of patients with a severe form of AP [10]. Interestingly, S1P synthetic analogue, fingolimod, may represent a novel therapeutic strategy in preventing AP and its complications [32, 33]. However, the question whether AP leads to disturbances in the metabolism of sphingolipids in the salivary glands, which are morphologically and functionally similar to the pancreas, remains unanswered.

In the presented study, we used a widely accepted animal model of cerulein-induced AP in order to examine sphingolipid metabolism in the salivary glands in the course of the disease. Cerulein is a cholecystokinin analogue that triggers AP via premature activation of trypsinogen within acinar cells, leading to excessive stimulation of pancreatic exocrine secretion [34]. In addition, there are no published data suggesting that cerulein stimulates the secretion of salivary amylase. Rats from our experiment were characterized by a significant increase in both amylase and lipase activities. These observations confirmed the development of AP and are consistent with the previously published results [35]. Furthermore, we noticed a considerable increase in CRP blood content in rats with cerulein-induced AP, which also confirms the previously published data. Increased CRP concentration is considered one of AP markers; it is therefore not surprising that in our experiment, we observed significantly elevated CRP concentration, as also evidenced by other researchers [36].

The most important and novel findings of our study were the observed changes in sphingolipid metabolism in the salivary glands in the course of AP and streptozotocin-induced diabetes. We narrowed our interests to two major types of salivary glands: the parotid and submandibular ones, which contribute up to 85% of unstimulated saliva flow (65% and 20% from the submandibular and parotid salivary glands, respectively) [37]. Surprisingly, we found no changes in the levels of particular sphingolipids in SMSG in the course of acute pancreatitis. In PSG, however, we observed significantly elevated levels of ceramide de novo synthesis precursors, sphinganine, and sphinganine-1-phosphate. The level of sphingosine, a downstream metabolite of ceramide, was also increased. On the other hand, S1P content remained decreased, which seems to contradict the elevated S1P level observed in the pancreatic tissue samples in the course of AP [11]. Finally, we demonstrated some changes in the content of particular sphingolipids in the salivary glands in the course of type 1 diabetes. In the sole paper on the aforementioned subject, Garbowska et al. [23] showed diversity in sphingolipid metabolism depending on the type of salivary gland [23]. Briefly, the cited authors showed increased S1P and SFA1P levels in streptozotocin-treated rats vs. control animals in the submandibular glands, while in the presented study, we found a decrease of the said sphingolipid particles. The same changes were noted in the parotid glands in which a significant upregulation of S1P, SFA, and SFO was observed, while in the study by Garbowska et al. [23], no changes were reported. Furthermore, we demonstrated considerably elevated levels of downstream metabolites of ceramide as well as SFO and S1P in PSG and decreased S1P content in SMSG. The parotid and submandibular glands represent two different types of metabolism: oxidative (PSG) and glycolytic (SMSG) [38]. The latter appears important given a different response pattern observed in some different tissues like skeletal muscles [12] and the salivary glands [22] in diabetic animals and at least partially explains the aforementioned differences.

Thus, we were the first to explore and describe the notion that acute pancreatic injury, both related to exocrine and endocrine functions, affects sphingolipid metabolism in the parotid, but not submandibular, salivary glands. This is in contrast to streptozotocin-induced diabetes, which could be associated with changes in the sphingolipid composition in both analyzed salivary gland types. Nevertheless, it should be highlighted that alterations in the concentration of particular sphingolipids in the salivary glands differ from those observed in pancreatic tissue samples. While some previously published reports proved several changes in sphingolipid metabolism in peripheral blood in the course of AP in human subjects, no studies have been performed to assess sphingolipid changes in the salivary glands of diabetic patients. Thus, before the introduction of our results into the clinical practice, further studies on the subject are required.

## Figures and Tables

**Table 1:** Effect of acute pancreatic injury on changes in amylase and lipase activities, C-reactive protein, glucose, and insulin concentrations in serum (mean ± SD).

Parameter	C	AP	DMt1
Amylase (IU)	1148 ± 73	2831 ± 387^∗^	1072 ± 54
Lipase (IU)	98 ± 17	654 ± 275^∗^	110 ± 29
CRP (mg/L)	7.6 ± 3.2	84.3 ± 18.2^∗^	10.2 ± 4.8
Glucose (mg/dL)	103.2 ± 17	115.4 ± 28	525 ± 46^∗#^
Insulin level (*µ*U/mL)	4.8 ± 1.2	4.4 ± 2.6	nd

C: control; AP: cerulein-induced acute pancreatitis; DMt1: streptozotocin-induced diabetes mellitus type 1. ^∗^*p* < 0.05 compared with the C group, ^#^*p* < 0.05 compared with the AP group. CRP: C-reactive protein; nd: non detected.

**Table 2:** Effect of acute pancreatic injury on sphingolipid concentration in the submandibular salivary gland (mean ± SD) (pmol/mg, except for sphingomyelin which is expressed in nmol/g).

	C	AP	DMt1
SM	1528.85 ± 71.489	1556.69 ± 123.471	1108.07 ± 82.822^∗#^
CER	227.85 ± 53.82	210.61 ± 35.967	239.05 ± 90.151
SFO	3.8 ± 0.44	4.42 ± 1.145	5.06 ± 1.385^∗^
SFA	0.31 ± 0.037	0.32 ± 0.073	0.16 ± 0.038^∗#^
S1P	0.28 ± 0.117	0.23 ± 0.065	0.16 ± 0.038^∗#^
SFA1P	0.26 ± 0.042	0.32 ± 0.091	0.16 ± 0.046^∗#^

C: control; AP: cerulein-induced acute pancreatitis; DMt1: streptozotocin-induced diabetes mellitus; ∗: vs. C (*p* < 0.05); #: vs. AP (*p* < 0.05). SM: sphingomyelin; CER: ceramide; SFO: sphingosine; SFA: sphinganine; S1P: sphingosino-1-phosphate; SFA1P: sphinganine-1-phosphate.

**Table 3:** Effect of acute pancreatic injury on sphingolipid concentration in the parotid salivary gland (mean ± SD) (pmol/mg, except for sphingomyelin which is expressed in nmol/g).

	C	AP	DMt1
SM	1510.02 ± 236.537	1482.35 ± 197.289	1119.82 ± 212.009^∗#^
CER	411.89 ± 105.358	361.01 ± 68.006	67.05 ± 10.754^∗#^
SFO	3.67 ± 0.354	4.64 ± 0.743^∗^	4.52 ± 0.531^∗^
SFA	0.13 ± 0.033	0.21 ± 0.037^∗^	0.31 ± 0.065^∗#^
S1P	0.17 ± 0.071	0.09 ± 0.046^∗^	0.26 ± 0.074^∗#^
SFA1P	0.1 ± 0.012	0.17 ± 0.051^∗^	0.13 ± 0.023^∗^

C: control; AP: cerulein-induced acute pancreatitis; DMt1: streptozotocin-induced diabetes mellitus; ∗: vs. C (*p* < 0.05); #: vs. AP (*p* < 0.05). SM: sphingomyelin; CER: ceramide; SFO: sphingosine; SFA: sphinganine; S1P: sphingosino-1-phosphate; SFA1P: sphinganine-1-phosphate.

## Data Availability

All data achieved during our experiment are included in the tables attached to the manuscript.
